# Seminal plasma microbiomes, sperm parameters, and cryopreservation in a healthy fertile population

**DOI:** 10.3389/fmicb.2024.1401326

**Published:** 2024-09-30

**Authors:** Longlong Fu, Yuanlei Lou, Ying Guo, Fang Zhou, Jing Ma, Shusong Wang, Yiqun Gu, Bin Fu, Wenhong Lu

**Affiliations:** ^1^Reproductive Health Research Centre, National Research Institute for Family Planning, Beijing, China; ^2^Jiangxi Provincial Key Laboratory of Urinary System Diseases, Institute of Urology, The First Affiliated Hospital, Jiangxi Medical College, Nanchang University, Nanchang, Jiangxi, China; ^3^Hebei Key Laboratory of Reproductive Medicine, Hebei Reproductive Health Hospital, Shijiazhuang, China

**Keywords:** seminal plasma microbiomes, fertile, sperm parameters, sperm cryopreservation and thawing, fertility

## Abstract

**Background:**

Recent advances in microbiome research have revealed the presence of diverse microbial communities in human tissues previously thought to be sterile. The present study delves into the emerging field of seminal plasma microbiomics, examining the relationship between semen microbes and semen parameters and post-freezing tolerance.

**Methods:**

The study involved a cohort of healthy fertility males and microbial genome analysis using 16S rRNA to characterize the microbial diversity of seminal plasma. Microbial diversity analysis identified unique amplicon sequence variants (ASVs) and genera dominant in seminal plasma. Spearman’s correlation coefficient was used to assess the relationship between flora and semen parameters. A paired t-test was used to compare the changes in microbiome expression in seminal plasma before and after cryo-resuscitation.

**Results:**

The relevant results show that the top five phyla in terms of abundance of seminal plasma microbiome were *Firmicutes, Bacteroidota, Proteobacteria, Actinobacteriota*, and *Campylobacterota*. Spearman correlation analysis highlighted the association between specific microbial species and semen parameters, between *Porphyromonas_asaccharolytica* and sperm concentration. Microbial changed significantly after cryo-resuscitation, affecting taxonomic units such as *Campylobacter* and *Muribaculaceae*, and KEGG enrichment analyses, suggesting that metabolic pathways are associated with sperm freezing. *Eubacterium_coprostanoligenes* and *Eptoniphilus_duerdenii* exhibited a potential impact, while *Orynebacterium_tuberculostearicum* demonstrated a positive correlation with the recovery rate of progressive motile sperm.

**Conclusion:**

The semen of normal fertile individuals contains a microflora component that is closely related to semen quality, including the sperm’s ability to withstand freezing.

## 1 Introduction

In the human body, many tissues previously thought to be sterile have now been shown to harbor microbes (Banerjee and van der Heijden, 2023). These microorganisms and their genetic material, collectively referred to as the microbiome, can play an important role in human health and disease resistance through a variety of mechanisms. Recent studies have shown that the human microbiome is associated with a variety of disorders, including the urinary and reproductive systems (Altmäe et al., 2019; Juarez et al., 2022). In recent years, semen microbiomics has become an area of increasing interest to scientists. A growing body of evidence suggests that microorganisms found in semen are associated with sperm abnormalities, particularly indicators of sperm motility, mitochondrial function, and DNA integrity (Lundy et al., 2021; He et al., 2024). The semen microbiome also has important implications for the reproductive health of men and couples, and the health of future generations (Koedooder et al., 2019). Many studies have shown that the species composition of semen flora varies greatly from man to man (Lundy et al., 2021; He et al., 2024; Hou et al., 2013).

However, study of the semen microbiome is limited and underpowered, and more data are needed to support it. The method of semen sampling, location of sperm collection and laboratory aseptic environments limit the study of semen microbiome. This is because many studies have adopted substandard methods that do not ensure the sterility of the sampling site or personnel hand hygiene (Eisenhofer et al., 2019). The analytical methods of microbiomics also limit the information. In the 1990s, the earliest and most traditional studies of semen bacteria were based on semen petri dish cultures. Since then, PCR or qPCR techniques have been used, which are more sensitive and allow for quantitative analysis. However, the disadvantage of this method is that it requires prior selection of the species of interest to be analyzed. This limitation was overcome with the entry of the bacterial 16S rRNA gene into the field of microbiome research: it provides extensive molecular characterization and quantification, eliminates the need for prior selection of species, and opens up a wide range of possibilities for subsequent bioinformatics analysis (Silva et al., 2023).

To the best of our knowledge, whether microorganisms in semen are associated with sperm freezing tolerance has not been studied. Therefore, we used prokaryotic 16S RNA to detect microbial diversity in seminal plasma from a healthy reproductive population and to investigate its correlation with sperm parameters and sperm freezing results.

## 2 Materials and methods

### 2.1 Ethics statement

This study was conducted with the approval of Human Subjects Ethics Committee of the National Research Institute for Family Planning (NRIFP2023024), and all the study participants provided written consent before participating in the study, agreeing to deliver their own anonymized information for future studies.

All semen samples were obtained at the Clinical Medicine Center and Human Sperm Bank of National Research Institute for Family Planning, Beijing China.

### 2.2 Clinical study design and subjects

In order to reduce the influence of other factors, so we chose healthy population of fertile men population for the study. To ensure that research participants are representative of the population, we set relevant inclusion and exclusion criteria. Inclusion Criteria: (i) healthy young men of fertile couples whose spouses were pregnant within 12 months, according to WHO manual criteria; (ii)completion of a physical examination, blood, urine and semen tests and a questionnaire; (iii)examined between 06/2022 and 06/2023. Exclusion Criteria: (i) underlying genetic or other diseases (sexually transmitted diseases, reproductive tract infections, cardiovascular diseases, obesity) that severely affect male fertility; (ii)long-term exposure to radioactive rays and toxic substances; (iii)taking medications (antibiotics, immunosuppressants, systemic corticosteroids, or chemotherapeutic drugs) in the last 3 months; (iV)semen infections, urinary tract infections, and sexually transmitted diseases; (v) smoking cigarettes, using drugs, and abusing alcohol.

### 2.3 Methods of semen collection and routine semen analysis

Candidates were asked to collect a semen sample after 2–7 days of abstinence, by masturbation into a sterile container prepared by the sperm bank. Prior to sample collection, subjects are instructed on the procedures to be followed to prevent sample contamination, to wash their hands with soap 2–3 times, the penis is first washed with warm soapy water, especially the glans and the coronal sulcus, then wiping with 75% alcohol 2–3 times. The semen was ejaculated directly into a sterile glass container, by avoiding contact with the sterile inner wall of the container. Freshly collected semen was used for routine semen clinical testing, semen bacterial culture and microscopic examination. The semen sample was placed in an incubator at 37°C immediately after collection and after complete liquefaction, routine semen analysis was performed according to the guidelines of World Health Organization 5th edition (World Health Organization, 2010). Semen volume (SV), sperm concentration (SC), and percentage of progressive motile spermatozoa (PR) were recorded. Semen (500 μL) was taken for microbiome analysis and stored at −80°C before processing.

### 2.4 Semen cryopreservation protocols

The sperm freezing process was conducted completely in accordance with the routine operations performed in the sperm bank. A mixture of glycerol, egg yolk and citrate (GEYC) was selected as cryoprotectant and frozen as recommended by the WHO manual (World Health Organization, 2010). Before sperm cryopreservation, the GEYC cryoprotectant was thawed at 25°C and then dropwise to the semen in a ratio of semen: protectant at 2: 1. After mixing, it was incubated at 30–35°C for 5 min and then slowly frozen using a freezable programmer (Air Liquide, France): from 20°C to −6°C at a rate of 1.5°C per minute, then cooled to −100°C at 6°C per minute and frozen at −100°C for 30 min. Finally, the sample tubes were transferred to liquid nitrogen where they were stored for at least 6 months. The recovery rate of progressive motile spermatozoa (PRR) was recorded. Similarly, 500 μL of post-thaw semen was used for microbiome analysis.

### 2.5 Genomic DNA extraction and purification

For fresh and cryo-resuscitation semen, specimens were centrifuged at 16,000 g for 6 min at 37°C to obtain sperm-free seminal plasma, according to WHO manual standards. Seminal plasma was separated and stored in separate tubes for microbiomic analysis of seminal plasma.

In this study, the negative controls were meticulously implemented to minimize the risk of contamination, including potential sources: glass receptacle, both unopened and opened freezing tubes with recommendations from a previous study (Nossa et al., 2010). Genomic DNA was meticulously extracted from the samples using the MagPure Soil DNA LQ Kit (Magan, United States), following the provided instructions. Subsequently, NanoDrop 2000 (Thermo Fisher Scientific, United States) and agarose gel electrophoresis were used to detect the concentration and purity of microbial DNA. The DNA obtained securely stored at −20°C. The extracted genomic DNA served as the foundation for PCR amplification of the bacterial 16S rRNA gene, employing specific primers with Barcode and the Takara Ex Taq high-fidelity enzyme. The broadly utilized primers, 343F (5’-TACGGRAGGCAGCAG-3′) and 798R (5’-AGGGTATCTAATCCT-3′), amplified either the V3-V4 or V4-V5 variable regions of the 16S rRNA gene, facilitating subsequent bacterial diversity analysis (He et al., 2024).

### 2.6 Library construction and sequencing

The PCR amplification products were detected through agarose gel electrophoresis, followed by purification with AMPure XP beads. Subsequently, the purified products served as templates for a second round of PCR amplification. Following the second PCR, the products were once again purified with AMPure XP beads. The purified products were quantified by with the Qubit system, and the concentrations were adjusted to meet the sequencing requirements. The sequencing process employed the Illumina NovaSeq 6,000 sequencing platform, generating double-ended reads with a length of 250 base pairs. Sequencing procedures were meticulously executed by Shanghai OE Biotechnology Co. (Shanghai, China), ensuring the acquisition of high-quality sequencing data.

### 2.7 Bioinformatic analysis

Sequencing and data analysis were performed by Shanghai OE Biomedical Technology Co. To collect the raw data, we first used Cutadapt software to cut out the primer sequences from the raw data sequences. Then the qualified double-ended raw data from the previous step were analyzed by DADA2 (Callahan et al., 2016) and QIIME2 (Bolyen et al., 2020) according to the default parameters for quality control, including quality filtering, noise reduction, splicing, and chimera removal, etc., to obtain representative sequences and ASV abundance. The representative sequences of each ASV were selected by the QIIME 2 software package, and all representative sequences were compared with the Silva (version138) database.^1^ Species comparison annotations were analyzed from the default parameters of the q2-feature-classifier software. Alpha and beta diversity analyses were performed using QIIME 2 software and the Chao (Chao and Bunge, 2002) index; the Shannon (Hill et al., 2003) indices were used to assess the alpha diversity of the samples. By using the unweighted Unifrac distance matrix calculated by R, unweighted Unifrac principal coordinate analysis (PCoA) was performed to assess the beta diversity of the samples. ANOVA/Kruskal Wallis/T test/Wilcoxon statistical algorithm based on the R package were used for analysis of variance. Species abundance spectra were variance analyzed by using LEfSe.

Thereafter, the metabolic pathways in the KEGG database^2^ were utilized to annotate the different metabolites to identify the pathways associated with these metabolites. Pathway enrichment analysis was performed using the Python package scipy.stats^3^ and the biological pathways associated with sperm cryo-resuscitation were identified by Fisher’s exact test.

### 2.8 Statistical analysis

Statistical analysis was performed on Prism 5 software. Data were analyzed by the D’Agostino-Pearson normality test. Paired t-test was used to compare the differences between groups. Spearman’s correlation coefficient was used to assess the associations between flora and semen parameters. *p*-values less than 0.05 were considered statistically different. The data are expressed as mean ± SD.

## 3 Results

### 3.1 Demographic and semen parameters

A total of 28 cases of fertile males (within less than 12 months of pregnancy waiting time) were included in this study. And these volunteers were all Han Chinese. Semen samples from the study participants were collected for analysis within 1–3 months after successful delivery by their female partner. The mean age of the 28 men was 28.1 ± 2.9 year, BMI 22.5 ± 2.5, duration of abstinence was 4.4 ± 1.4 days, and the values of their semen parameters: semen volume (SV) 4.4 ± 1.1 (ml), sperm concentration (SC) 74.6 ± 22.6*10^6^/ml, and percentage progressive motile spermatozoa (PR) 56.5 ± 7.8%. While after cryo-resuscitation, the recovery rate of progressive motile spermatozoa (PRR) in the fertile population of 28 cases was 67.8 ± 16.8%.

### 3.2 Structure and diversity of microflora in seminal plasma in fertile populations

**Figure 1:**
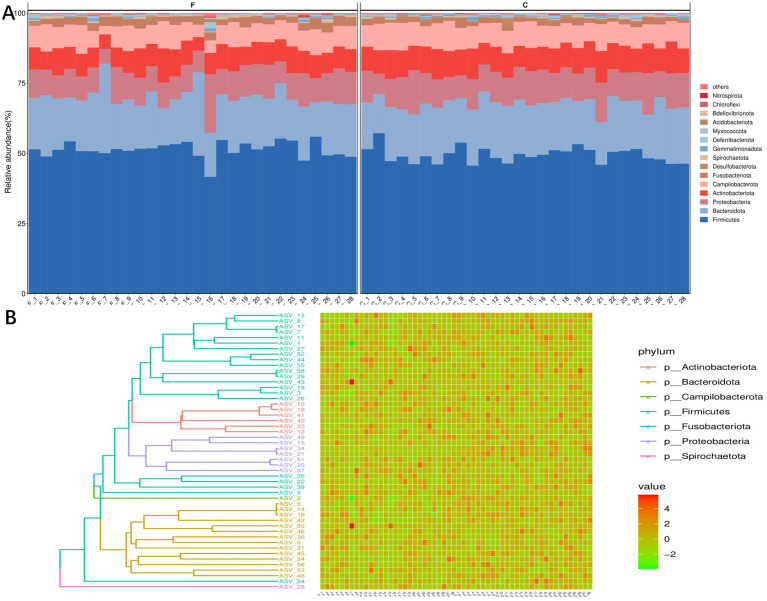
Distribution and diversity analysis of semen colonies of fertile men. **(A)** Histogram of the community structure of the 28 semen samples, before and after cryo-resuscitation (TOP15 is shown as an example). **(B)** TOP50/100 species evolutionary tree and ASV abundance plot. On the left is the evolutionary tree diagram with phylum as the gate information; on the right is the abundance plot corresponding to the abundance of ASVs in each sample on the left.

A total of 6,826 unique amplicon sequence variants (ASVs) were identified. We analyzed the dominant genera in the semen, and the results are shown in Figure 1. The results of the histogram of the distribution of the TOP15 species ranked by AVS abundance in seminal plasma before and after cryo-resuscitation are shown in Figure 1A. And the abundance of each ASV was counted, and the top 50 ASVs with the most tags (the most abundant ones) were selected to construct an evolutionary tree (Figure 1B). At phylum level of classification, we found that the top five phyla in terms of abundance of bacteria were *Firmicutes, Bacteroidota, Proteobacteria, Actinobacteriota, and Campylobacterota*. Next, we analyzed the dominant genera and the results show that *Fenollaria, Campylobacter, Peptoniphilus, Negativicoccus, Prevotella, Porphyromonas, Muribaculaceae, Bacteroides, Mobiluncus,* and Var*ibaculum* were the top 10 genera in relative abundance at the genus level.

### 3.3 Distribution of microbial community in seminal plasma in fertile populations

**Figure 2:**
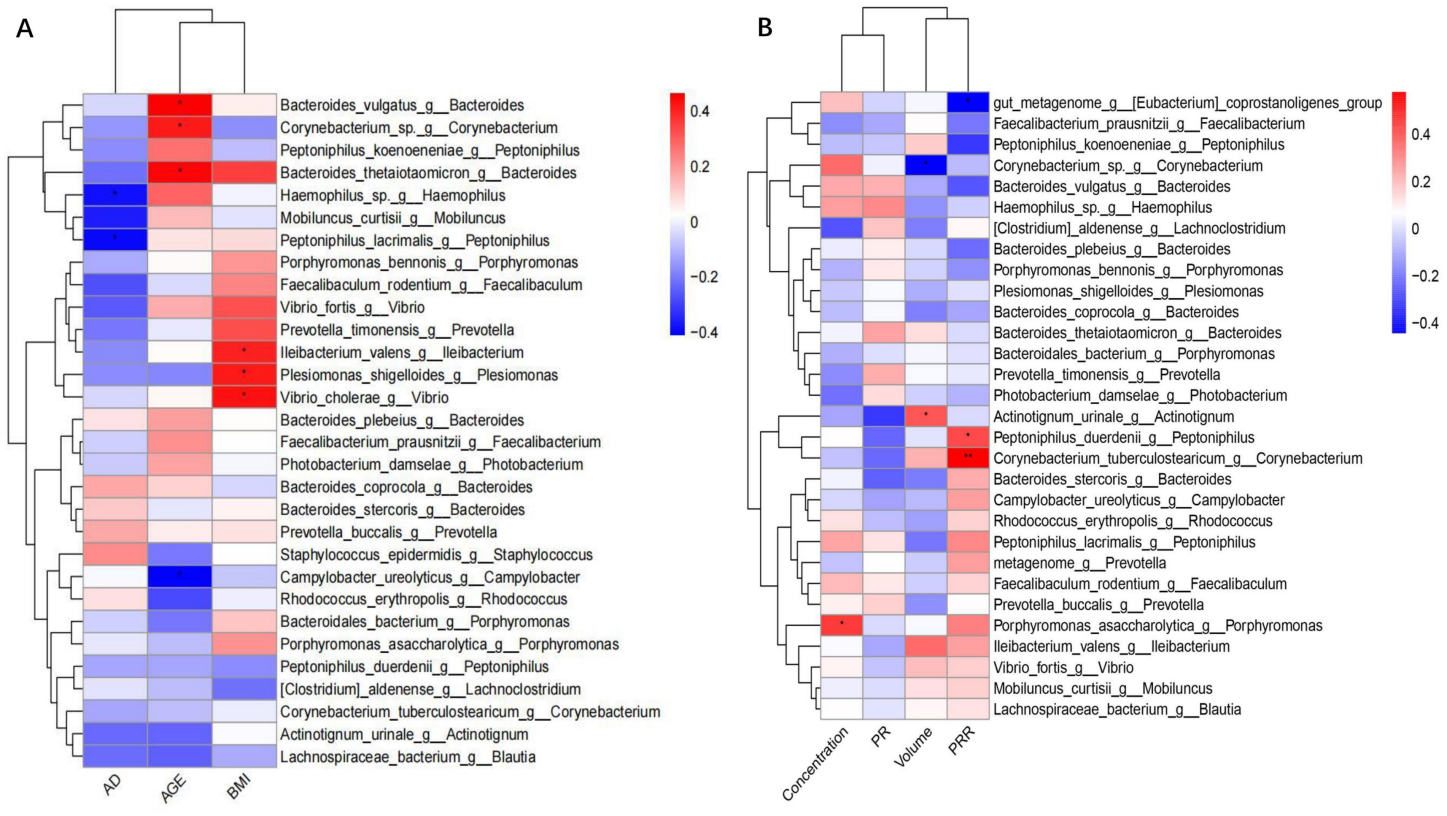
Legend of correlation between bacterial flora data with demographic and sperm parameters. **(A)** Heatmap of the correlation between bacterial flora and demographic. **(B)** Heatmap of the correlation between bacterial flora and semen parameters.

Our results showed that the distribution of microorganisms in the fertile population was correlated with age and body mass index (BMI), but not with the number of abstinence days (AD) (Figure 2A). *Corynebacterium* and *Bacteroides* were positively correlated with age, with Spearman’s coefficients of 0.42 and 0.44, respectively. *Bacteria* correlated with BMI included *Plesiomonas Ileibacterium, Bacteroides* and *Vibrio* with Spearman’s coefficients of 0.48, 0.41, 0.38 and 0.48, respectively. We identified microbial species demonstrating significant associations with key semen parameters values (Figure 2B). *Porphyromonas_asaccharolytica* was positively correlated with sperm concentration (r = 0.46, *p* = 0.015). Similarly, *Actinotignum urinale* was positively correlated with semen volume (r = 0.4055, *p* = 0.032). Conversely, *Corynebacterium* displayed a negative correlation with semen volume (r = −0.4437, *p* = 0.018).

### 3.4 Correlation between microorganisms and human sperm freezing resistance

Analysis of correlation results showed that *Eubacterium coprostanoligenes* (r = −0.4412, *p* = 0.019) and *Peptoniphilus_duerdenii* (r = −0.3280, *p* = 0.022) were negatively correlated with PRR. In contrast, *Corynebacterium_tuberculostearicum* was positively correlated with PRR (r = 0.5789, *p* = 0.001) (Figure 2B).

**Figure 3:**
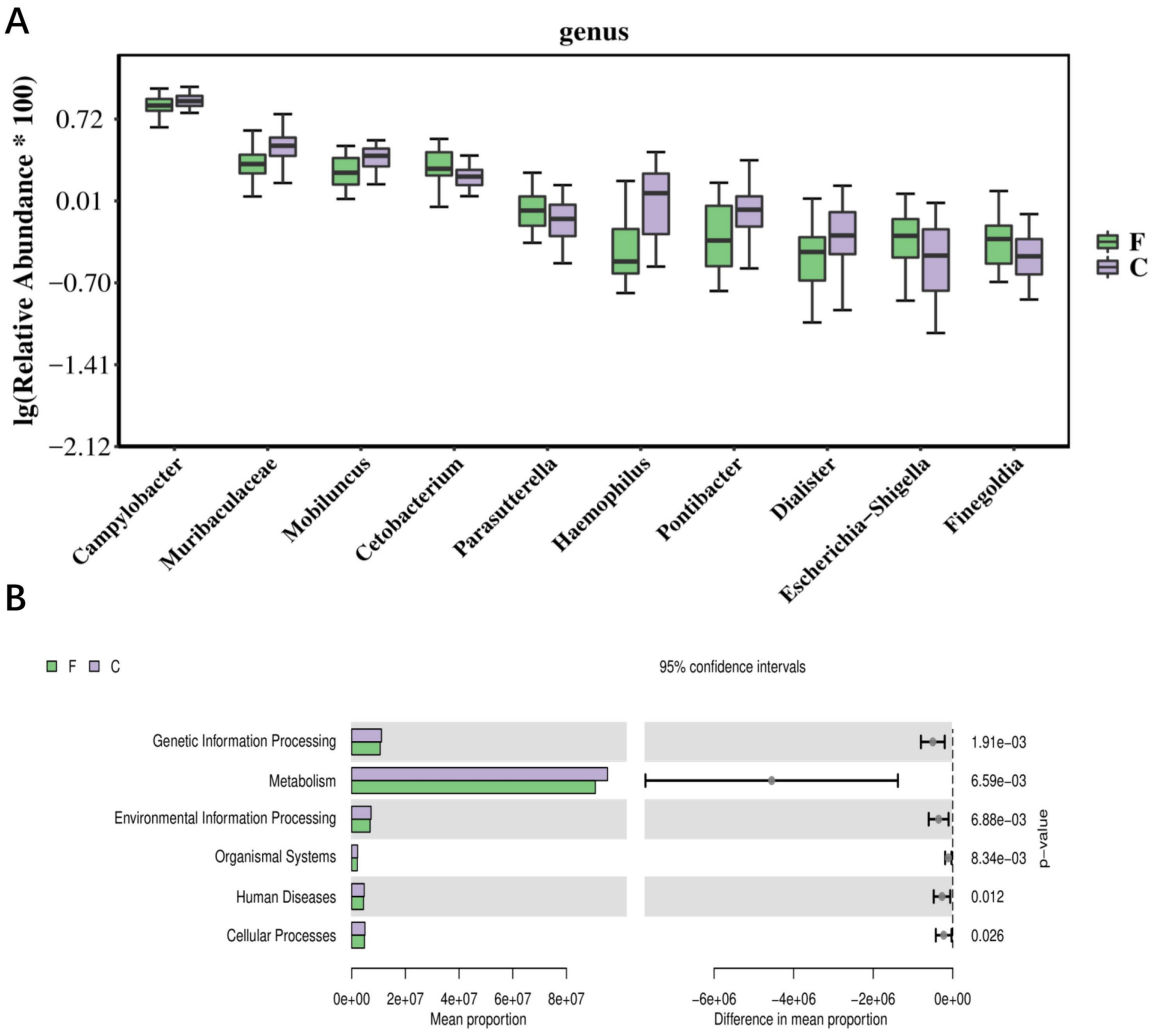
The correlation between bacterial flora data and the sperm cryopresevation **(A)** Top10 boxplot of the abundance of individual bacterial differential species in semen before and after cryo-resuscitation. **(B)** Bar graph of KEGG differential results, with bars showing the average abundance of pathways in each group. F, fresh semen; C, Cryo-resuscitated semen.

Significant changes in the abundance of specific taxa occurred before and after cryo-resuscitation: these included *Campylobacter, Muribaculaceae, Morbillus, Cetobacterium, Paracoccidioides, Haemophilus, Pontiobacterium, Togglecoccus, Shigella*, and *Pseudomonas fine-golden glucose* (Figure 3A). Based on these differences, the results of KEGG analyses showed that there may be correlations with sperm freezing in pathways related to environmental information processing, genetic information processing, human diseases, metabolism, and organismal systems (Figure 3B). Differential analysis of these microbial populations lays the foundation for a comprehensive study of the biological responses of bacteria during cryopreservation, providing a more multidimensional interpretation.

## 4 Discussion

According to the new report released by the World Health Organization: from 48.5 million to 72.4 million couples suffer from infertility, and the global prevalence of infertility in couples of childbearing age ranges between 12.6 and 17.5 per cent (about one in six) (Hill et al., 2003). With research progress, researchers are increasingly recognizing a variety of medical and social phenomena as potential contributors to infertility. The semen microbiome is a tiny and diverse ecosystem that is believed to have a significant impact on male reproductive health (Cox et al., 2022). Unlike as in previous studies (Altmäe et al., 2019; Osadchiy et al., 2024), a group of fertile males was selected for this study in strict adherence to the criteria set forth in the World Health Organization manual (Silva et al., 2023). We aim to provide a repository of reference data on the microbial composition of male semen, with the ultimate goal of elucidating potential associations between the distribution of semen microbiota and male reproductive health. We seek to increase the relevance and applicability of our findings to the broader context of fertile males.

The presence of a symbiotic microbial community in the semen of reproductively healthy males is well-established and, in our study, we utilized the 16S rRNA approach. Our observation of significant variations in microbial colonies among different males suggests that these communities are not only unique but also highly diverse, possibly representing distinct and independent microbial ecosystems. Studies, such as those conducted by Willén et al. (1996), have emphasized that different detection methods can yield disparate results. While acknowledging the existence of some systematic biases in genomic DNA template analysis, we want to underscore that our results, particularly those pertaining to microbial distribution and diversity analysis, indeed reflect the true state of the microbial landscape in semen (Willén et al., 1996) since we have mitigated these biases by controlling for appropriate sample sequencing quantities and adhering to rigorous data analysis protocols. Our findings stand as reasonable and reliable representations of microbial diversity and distribution in semen, providing a solid foundation for further in-depth exploration of the seminal microbiome.

Apart from the method of detection, there are many factors that influence the distribution of flora, which may include obesity, race, diet, sexual lifestyle, antibiotic etc., and many of these associations need to be fully investigated (Kim et al., 2020). Consistent with previous literature, we identified a rich microbiome from semen (Osadchiy et al., 2024; Hopkins et al., 2001). *Fenollaria, Campylobacter, Peptoniphilus, Negativicoccus, and Prevotella* are overrepresented in the semen of fertile males, suggesting a distinctive contribution from upstream anatomical locations such as the seminal vesicles, prostate, or testes. Our data show found that *corynebacterium* and *vibrio (Bacteroides)* were positively associated with age in fertile populations. Microorganisms associated with body mass index include *Plesiomonas Ileibacterium, Bacteroides,* and *Vibrio*. *Porphyromonas_asaccharolytica (Porphyromonas)* was positively associated with sperm concentration and *Actinotignum urinale (Actinotignum urinale)* was positively associated with semen volume; on the contrary, *Corynebacterium (Corynebacterium)* was negatively associated with semen volume. A study by Hou et al. (2013) also found: that most of the samples from sperm donors and infertility patients had a large number of different species of bacteria and that sperm quality was significantly negatively correlated with the presence of anaerobic cocci. A previous study by Lundy et al. (2021) also showed that the abundance of *anaerobic coccobacilli precursors* was inversely correlated with sperm concentration and that *Pseudomonas aeruginosa* was directly correlated with the total number of motile spermatozoa. And these data collectively support the existence of an intrinsic testicular microbiota that may play a role in spermatogenesis (Chen et al., 2018). This result, contributes to male fertility. For example, semen quality can be improved by modifying the microflora. In addition, testing of the microflora may be part of a male reproductive health assessment. Next, we explored the correlation of the microbiome with semen parameter values in healthy fertile men, which may merit further study.

Sperm cryopreservation is currently the “gold standard” for male fertility preservation and is one of the basic elements of assisted reproductive technology (Baud et al., 2019; Drechsel et al., 2023). However, owing to the multiple factors involved in cryo-resuscitation, such as ice crystal formation, cold shock and, oxidative stress, it can lead to structural and functional damage to spermatozoa: decreased sperm viability and quality (Fu et al., 2019). To the best of our knowledge, no studies have been conducted on whether microorganisms in semen are associated with sperm freezing tolerance. Therefore, we investigated the relevance of microbiota during human sperm freezing. It was found that *Eubacterium coprostanoligenes_group* and *eptoniphilus_duerdenii* were highly correlated with post-thaw sperm quality, while *Corynebacterium_tuberculostearicum* were positively correlated with PRR. The combined KEGG analysis of semen differential flora before and after cryo-resuscitation showed that metabolism-related biological processes be altered during sperm freezing. This is consistent with the results of previous studies (Dai et al., 2019; Ali et al., 2023). The mechanisms of protection are not fully understood, but they may be related to the antioxidant products exerted in the extracellular environment (Fu et al., 2024). These mechanisms are now mainly likely to focus primarily on oxidative stress and the production of biofilms. Antioxidants are produced, which help to reduce the oxidative stress that increases during freezing and thawing (Kilama et al., 2024). The results of this study should provide a new direction for our research.

## 5 Limitation

Our study wanted to show that there may be certain microbiome families that harmoniously dwell in semen. The study collected data on a healthy fertile population, which is in strict accordance with the standards of the World Health Organization manual. And to the best of our knowledge, the correlation between flora and sperm freezing was explored for the first time. However, this was a single-institution study with a small number of subjects, 28 semen samples from 28 fertile men. Larger multi-institutional longitudinal studies are needed to minimize the risk of data overfitting and to validate these results. Whether dysbiosis associated with spermatogenesis is simply a “canary in the coal mine” for overall health, or whether the differences demonstrated here are truly causal and specific to reproductive health, remains to be further clarified. Further studies (including metaculture histology or gene biology) are needed to validate mechanistic pathways and identify candidate therapeutic targets before clinical translation.

## 6 Conclusion

The prevalence of *Firmicutes, Bacteroidota, Proteobacteria, Actinobacteriota* and *Campylobacterota* in semen and spearman correlations highlighted the association between specific microbial species and semen parameter values, between *Porphyromonas_asaccharolytica* and sperm concentration. Notably, microbial landscapes changed significantly after cryo-resuscitation, affecting such taxonomic units as *Campylobacter* and *Muribaculaceae*, and KEGG enrichment analyses, suggesting that metabolic pathways are associated with sperm freezing. *Eubacterium coprostanoligenes*_and *eptoniphilus_duerdenii* were less related than *orynebacterium_tuberculostearicum*, which were demonstrated a positive correlation with the recovery rate of progressive motile spermatozoa.

## Data Availability

The data presented in the study are deposited in the NCBI Sequence Read Archive (http://www.ncbi.nlm.nih.gov/Traces/sra), accession number PRJNA1159181.

## Footnotes

^1^https://www.arb-silva.de/

^2^https://www.kegg.jp/kegg/pathway.html

^3^https://docs.scipy.org/doc/scipy/
